# Praying until death: revisiting three delays model to contextualize the socio-cultural factors associated with maternal deaths in a region with high prevalence of eclampsia in India

**DOI:** 10.1186/s12884-019-2458-5

**Published:** 2019-08-28

**Authors:** Md Illias Kanchan Sk, Balram Paswan, Ankit Anand, Nasim Ahamed Mondal

**Affiliations:** 1 Department of Population Policies and Programmes, International Institute for Population Sciences, Govandi Station Road, Deonar, Mumbai, 400 088 India; 2 Institute for Social and Economic Change, Bangalore, India; 3 National Institute for Research in Reproductive Health, ICMR, Mumbai, India

**Keywords:** Three delays model, maternal deaths, facility-based, community-based, socio-cultural factors, verbal autopsy

## Abstract

**Background:**

A disproportionately high proportion of maternal deaths (99 percent) in the world occur in low and middle income countries, of which 90 percent is contributed by Sub-Saharan Africa and South Asia. This study uses the effective "Three Delays" model to assess the socio-cultural barriers associated with maternal mortality in West Bengal, India.

**Methods:**

It was a retrospective mixed methods study, which used facility-based as well as community-based approaches to explore factors associated with maternal deaths. We reviewed 317 maternal death cases wherein a verbal autopsy technique was applied on 40 cases. The Chi-square test (χ2) and multivariable logistic regression model were employed to accomplish the study objectives.

**Results:**

The delay in seeking care (Type 1 delay) was the most significant contributor to maternal deaths (48.6 percent, 154/317). The second major impacting contributor to maternal deaths was the delay in reaching first level health facility (Type 2 delay) (33.8 percent, 107/317), while delay in receiving adequate care at the health facility (Type 3 delay) had a role in 18.9 percent maternal deaths. Women staying at long distance from the health facilities have reported [AOR with 95 % CI; 1.7 (1.11-1.96)] higher type 2 delay as compared to their counterparts. The study also exhibited that the women belonged to Muslim community were 2.5 times and 1.6 times more likely to experience type 1 and 2 delays respectively than Hindu women. The verbal autopsies revealed that the type 1 delay is attributed to the underestimation of the gravity of the complications, cultural belief and customs. Recognition of danger signs, knowledge and attitude towards seeking medical care, arranging transport and financial constraints were the main barriers of delay in seeking care and reaching facility.

**Conclusions:**

The study found that the type-1 and type-2 delays were major contributors of maternal deaths in the study region. Therefore, to prevent the maternal deaths effectively, action will be required in areas like strengthening the functionality of referral networks, expand coverage of healthcare and raising awareness regarding maternal complications and danger signs.

## Background

Every minute a woman dies as a result of pregnancy and childbirth-related complications [1]. Recent WHO estimates show that pregnancy and child-birth related complications leads to more than 300,000 maternal deaths worldwide [2]. A disproportionately high proportion of these deaths (99 percent) occur in low- and middle-income countries (LMICs), of which 90 per cent is contributed by Sub-Saharan Africa and South Asia [2, 3]. According to the latest WHO estimates, 45000 maternal deaths occurred during 2015 in India and this figure drives India into the home of the second largest number of maternal deaths after Nigeria [1]. The level of maternal mortality ratio (MMR) in 2015 stood at 130 deaths per 100,000 live births which was far cry from 100 per 100,000 live births as per the Millennium Development Goal (MDG-5) for 2015 [1, 2]. Most of the maternal deaths (80-85%) in developing nations including India can be attributed to direct obstetric causes (haemorrhage, sepsis, complications of abortion, hypertensive disorders) [3, 4]. Timely treatment and management of pregnancy complications is the key to prevent maternal mortality and reduce the gap in maternal deaths between developed and developing countries [5]. The “three delays model” concept provided by Thaddeus and Maine (1990) [6] has proven as a useful and widely accepted framework to account for the delay in management for obstetric emergencies and its role in maternal mortality [7, 8]. The three delays model is widely considered as a comprehensive approach for examining barriers to seek obstetric care and prevent maternal mortality [7, 9]. The model explores what, why and how maternal deaths occurred. The role of the three delays model in maternal mortality was documented in several previous studies in Indian and global context. Studies have observed and reported delays in majority of the maternal death cases [3, 10–12]. This model helps to determine the factors associated with the community as well as health service levels which contribute to pregnancy and childbirth-related deaths. The findings will lead to formulation of strategies for preventive measures.

The study area, West Bengal, located in eastern part of India, comprises of 24.9 million women in reproductive age group (15-49 years) has 55 percent antenatal care coverage in the first trimester, 74 percent institutional delivery rate and 61 percent postnatal care coverage [13, 14]. A vast majority of maternal deaths in our study area were attributed to direct causes, particularly to eclampsia. Previous studies conducted in our study region reported that more than one-third maternal deaths in West Bengal occurred due to eclampsia, with a case fatality rate, higher than the average rate and a high incidence of Eclampsia [10, 15, 16]. The “three delays model” seems to be an appropriate mechanism for identifying the barriers on the ‘demand side’ and ‘provider or supply side’ which assess the maternal mortality [7]. These barriers may play a key role in the adverse outcomes of maternal health. Despite that the medical causes attributed to maternal deaths have been studied in detail in several studies, both at the global and national levels. But there is no sizeable literature available applying “three delays model” on the social factors that assume a paramount part in these deaths [6, 17]. The direct relationship between the occurrence of delay and severity of maternal outcome has never been systematically illustrated in previous studies. Therefore, the purpose of this study is to identify facility and community level factors that contributed to maternal deaths by employing three delays model. We also aimed to investigate the confounding factors associated with these delays.

## Methods

### Study Design

The design employed for this study was retrospective mixed methods. This retrospective study utilized the facility-based as well as community-based approaches to explore the maternal deaths. The study was conducted between November 2013 and October 2015 in two major tertiary level healthcare referral centres in West Bengal, India. During this two-year study period, a total of 317 maternal deaths were recorded. This study also conducted 40 verbal autopsies among family members of deceased women in the community.

### Study Settings

The health facilities that were selected were Malda Medical College and Hospital (MMC&H) and Medical College and Hospital, Kolkata (KMC&H), situated in the northern and southern part of West Bengal respectively. The referral hospitals conduct almost twenty-seven thousand deliveries annually (West Bengal Health Service, 2015). The selected health care units get the lion's share of complicated and high-risk patients from the neighboring townships and villages and also from other parts of India. Hence, the studied hospitals can bring up the representative sample and effective picture to comprehend the contribution of eclampsia to maternal mortality in the community as a whole.

### Data Collection Technique/Process

This study utilized primary and secondary data to accomplish the objectives of the study. We used hospital-based records of all maternal deaths (N=317) that occurred in the Department of Obstetrics and Gynecology of the selected hospitals. The details of all 317 maternal deaths during November 2013 and October 2015 were collected using facility-based maternal death review form (FBMDR). We reviewed the individual case sheet of the women, death registers, referral letters, medical records, Bed Heat Tickets (BHTs) and previous antenatal care records to retrieve the data related to socio-demographic profile and clinical profile of the deceased women.

As far as primary data was concerned, we conducted 40 verbal autopsies (including 20 from eclamptic deceased women and 20 from non-eclamptic deceased cases) in the community. The 40 cases were purposively selected from the 317 maternal deaths occurred in the studied hospitals. We interviewed those people who had witnessed any stage during the process leading to death. Those people were mainly family members, neighbours and relatives. In some cases, both sets of relatives – from parent’s side and in-law’s side were also interviewed together. We used the residential address maintained in the selected hospitals to reach the respondents. The tool used was maternal mortality questionnaire (unstructured and semi-structured schedules), which incorporated the NRHM-validated Verbal Autopsy Questionnaire (VAQ). Most of the interviews were conducted in Bengali (regional language) but few interviews were also done in Hindi (official language of India). The written informed consent was obtained from all family members of deceased women and also from the competent hospital authorities to conduct the study. The whole qualitative interview process took 57 days to finish and each interview took almost 40 minutes.

### Data compilation and analysis

Descriptive statistics were utilized to present the socio-demographic and health-seeking behaviours of deceased women. The cross tabulations with Chi-square test (χ2) was used to show the association between various attributes. The multivariable logistic regression model was applied to estimate the odds ratio with 95% confidence intervals and p-value less than 0.05 was considered statistically significant. The data analyses were performed with the statistical software STATA version 13.0 software. Most of the interviews were digitally recorded and later transcribed and translated into English. The English transcripts were imported into the -QSR N-Vivo 10 (categorized and analysed by adopting thematic approach).

### Operational Definition

i. Birth Preparedness and Complication Readiness (BPCR): It is a strategy of planning for safe delivery and awareness about the actions required in case of any emergency. In this study, we have included the strategies like preparation for normal birth by selecting skill birth attendants and place of delivery; preparation of essential items for delivery; knowledge of danger signs for mother and newborn; knowledge of where and to whom to go in case of an emergency; arranging access to funds and means for emergency transportation; and prior identification of blood donor under the Birth Preparedness and Complication Readiness (BPCR) category.
ii. Three Delays: The “three delays model” has been widely considered as a comprehensive approach for examining barriers to seek obstetric care and prevent maternal mortality. The model comprises the delay in deciding to seek appropriate care by individuals, family or both (Delay 1), delay in reaching an adequate health care facility (delay 2) and delay in receiving adequate care when a facility is reached (Delay 3) [6].
iii. Maternal Deaths: The WHO definition of maternal deaths was adopted to define the deaths during pregnancy and childbirth. A maternal death is defined as “the death of a woman while pregnant or within 42 days of the end of the pregnancy, irrespective of the duration and the site of the pregnancy, from any cause related to or aggravated by the pregnancy or its management, but not from accidental or incidental causes” [1].

## Results

### Distribution of the maternal deaths according to the levels of delay involved

Out of the 317 maternal deaths audited, 206 (64.9 per cent) had at least one type of delay (Table 1). Delay in seeking care (Type 1 delay) was the most significant contributor to maternal deaths and it led to 48.6 per cent (154/317) of the maternal deaths. The second major contributor to maternal deaths was the delay in reaching first level health facility (Type 2 delay) (33.8 percent, 107/317), while delay related to receiving the quality of medical care at the health facility (Type 3 delay) was observed in 60 women (18.9 percent). In most of the cases, more than one type of delay was present.

**Table 1:** Type and reason for delays identified for maternal deaths (*N*=317)

Type of Delay	Reason for the delay	No of women	Percentage (%)^*^
Type 1 Delay - Delay in Seeking Care (*n*=154, 48.6)	Unawareness of danger signs	99	31.2
	Illiteracy & Ignorance	62	19.6
	Delay in decision making	125	39.4
	No birth preparedness	96	30.3
	Beliefs and customs	14	4.4
	Non-availability of health care professional	11	3.5
	Any other	5	1.6
Type 2 Delay - Delay in reaching first level health facility (*n*=107, 33.8)	Delay in getting transport	55	17.4
	Delay in mobilizing funds	51	16.1
	Not reaching appropriate facility in time	99	31.2
Type 3 Delay - Delay in receiving adequate care in facility (*n*=60, 18.9)	Delay in initiating treatment	12	3.8
	Substandard care in hospital	27	8.5
	Lack of blood, equipment & drugs	44	13.9
	Any other	12	3.8
Any delay^#^		206	65.0

^*^More than one type of delay possible for each maternal death, presented in order of frequency, ^#^Categories are not mutually exclusive

### Reasons for delays at various levels

The main reason for the delay related to seeking care was late decision making (39.4 percent) (Table 1). Other two important reasons were unawareness of danger signs and no birth preparedness, which were found among 31.2 and 30.3 percent of cases respectively. Not reaching appropriate facility in time (n=99, 31.2 percent) was the most prevalent reason for Type 2 delay. In fifty-five (17.4 percent) maternal deaths, the second delay was ascribed to the unavailability of transport; in fifty-one deaths, it was due to financial constraints to avail transport and treatment. Lack of blood, equipment and drugs (44/60, 13.9 percent) were the factors in the maternal deaths in which third-type delay was noted.

### Causes of deaths and delays

**Fig. 1:**
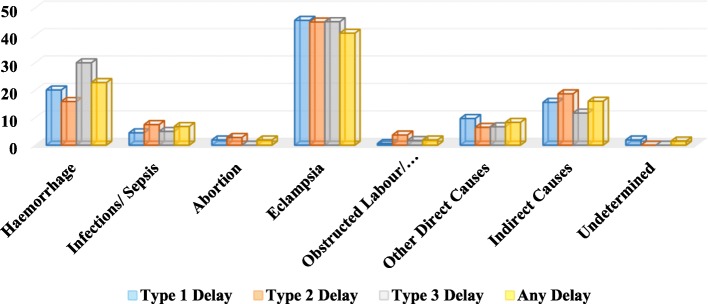
Causes of maternal deaths by major contributing delay (%)

Eclampsia was identified as the leading cause of maternal mortality in all three types of delays (Fig. 1). Among all the three delays, about 45 per cent of the maternal deaths had eclampsia. Type 3 delay, was maximum (30 per cent) in those who had haemorrhage as compared to other medical causes. In other direct causes, Type 1 delay (9.7 percent) appeared to be the main contributor in comparison to other delays, while Type 2 delay (18.7 percent) was observed as the main contributor in indirect causes of maternal mortality.

### Types of delays observed by selected socio-demographic and obstetric related characteristics

The occurrences of all three types of delays were more prevalent among women belonging to 20-24 years’ age group as compared to their counterparts (Table 2). The women who belonged to 20-24 years’ age group experienced 37, 39 and 50 percent of type 1, type 2 and type 3 delays respectively. Almost 90 percent of women from rural areas reported delays at different phases in contrast to 10 percent of urban cases. The delay in seeking care and delay in reaching health facilities were comparatively higher among Muslims than Hindus, while there was no difference in frequency (30) of type 3 delays owing to religious affiliation. Multigravida (68.2 percent) and multiparous (36.4 percent) were noted to be at highest risk of type 1 delay. The delay in reaching health facility was more common in second gravidas and primiparous women, which was found among 36.4 percent each in these groups. Patients who stayed far from the study hospitals experienced comparatively high proportions of type 2 and 3 delays. Type 2 and 3 delays were noted among almost half of the deceased women who resided more than 45 km away from the study hospitals. Of the 154 reviews in which type 1 delay was reported, delay in seeking care was present more frequent in women residing 25-49 km (37 percent) away from the study hospitals and followed by in those staying more than 49 km (one-third).

**Table 2:** Distribution of delays observed at different phases among deceased women by selected socio-demographic and obstetric related characteristics (N=317)

Characteristics	Types of delays					
	Type 1 (*n*=154)		Type 2 (*n*=107)		Type 3 (*n*=60)	
	*n*	%	*n*	%	*n*	%
Age at death						
<20	20	13.0	19	17.8	9	15.0
20-24	57	37.0	42	39.3	30	50.0
25-29	35	22.7	26	24.3	8	13.3
>29	42	27.3	20	18.7	13	21.7
Place of residence						
Rural	137	89.0	98	91.6	54	90.0
Urban	17	11.0	9	8.4	6	10.0
Religion						
Hindu	75	48.7	50	46.7	30	50.0
Muslim	79	51.3	57	53.3	30	50.0
Gravidity						
Primigravida	49	31.8	35	32.7	23	38.3
Gravida 2	41	26.6	39	36.4	17	28.3
>2 Gravida	64	41.6	33	30.8	20	33.3
Parity						
Nullipara	50	32.5	37	34.6	24	40.0
Primipara	48	31.2	39	36.4	17	28.3
Multipara	56	36.4	31	29.0	19	31.7
No of living Children						
0	66	42.9	47	43.9	27	45.0
1	39	25.3	34	31.8	15	25.0
>1	49	31.8	26	24.3	18	30.0
Distance between home & final hospital						
<25	46	29.9	21	19.6	17	28.3
25-49	57	37.0	32	29.9	14	23.3
>49	51	33.1	54	50.5	29	48.3

### Types of delays observed by health seeking behaviour and mode of delivery

All three types of delays were more commonly observed in women who had no ANC (Table 3). More than 90 percent women who did not receive any antenatal care experienced Type 1 delay. Similarly, Type 2 and 3 delays were present 70 and 60 percent respectively in null ANC attendance patients. The referred cases experienced more proportions of delays (all three types) as compared to non-referred women (more than 80 percent v/s less than 20 percent). Type 1 delay was more common in caesarean delivery mothers, at 42.6 percent for C-section delivery women compared with 30 percent for normal delivery women. Similar patterns of distributions were also noted in type 2 and 3 delays. In terms of period of admission, type 1, 2 and 3 delays were more frequent in patients who were admitted in postpartum/natal period.

**Table 3:** Distribution of delays observed at different phases among deceased women by health seeking behaviour and delivery related characteristics (*N*=317)

Characteristics	Type of delay					
	Type 1 (*n*=154)		Type 2 (*n*=107)		Type 3 (*n*=60)	
	*n*	%	*n*	%	*N*	%
No of ANC visits						
No	140	92.1	71	69.6	34	59.6
1-2	10	6.6	22	21.6	18	31.6
>2	2	1.3	9	8.8	5	8.8
Referral status						
Yes	129	83.8	93	86.9	49	81.7
No	25	16.2	14	13.1	11	18.3
Mode of delivery						
Undelivered	39	27.7	27	27.0	11	18.6
Vaginal/Vacuum/forceps	42	29.8	31	31.0	20	33.9
Caesarean section	60	42.6	42	42.0	28	47.5
Period of admission						
AN Period	49	31.8	28	26.2	17	28.3
Intrapartum	51	33.1	31	29.0	21	35.0
Postpartum/Natal	54	35.1	48	44.9	22	36.7

### Factors influencing the first and second delay

The binary logistic regression analysis in Table 4 shows that first delay was associated with age of death, parity and religion. Distance from home and the final hospital where deaths occurred and religion significantly influenced the second delay. Women belonged to Muslim community were 2.5 times and 1.6 times more likely to experience type 1 and 2 delays respectively than Hindu women. The result also shows that as the age of women and parity increases the likelihood for first delay also increases. Women who stayed farther away from the hospitals were more likely [AOR with 95 % CI; 1.7 (1.11-1.96)] to have the second delay as compared to the women who resided at shorter distances.

**Table 4:** Logistic Regression showing variables influencing the First Delay and Second Delay

Variables	Delay 1			Delay 2		
	OR	95% C.I.		OR	95% C.I.	
		Lower	Upper		Lower	Upper
Age©	1.81^*^	1.11	2.13	1.15	0.67	2.28
Religion						
Hindu®	1.00			1.00		
Muslim	2.48^***^	1.91	3.26	1.58^**^	1.28	1.95
Place of residence						
Rural®	1.00			1.00		
Urban	0.56	0.04	1.14	0.57	0.23	1.49
Parity©	1.95^**^	1.38	2.28	1.23	0.85	2.90
Gravidity©	1.31	0.65	3.21	0.85	0.04	3.91
Distance between home and final hospital (km)©	1.13	0.82	2.56	1.67^**^	1.11	1.96
ANC Visits						
Yes®	1.00			1.00		
No	1.57	0.45	2.21	2.07	1.09	3.36
Referral status						
Yes®	1.00			1.00		
No	1.95	0.84	2.99	1.24	0.25	2.72

*P* values- *** Significant at 1 percent; ** Significant at 5 percent; * Significant at 10 percent. (R)=Reference Category, ©=Continuous Variables

### Results of Verbal autopsies

#### Delay in seeking care

Out of 40 verbal autopsies, 35 women had delay in seeking care. The delay ranged between 2 hours to 3 days. This delay is attributed to underestimation of the gravity of the complications, cultural belief and customs and unfavourable experiences with the health system. Some have cited lack of money as one of the barriers and refusal to receive medical attention.

##### Underestimation of severity of danger sign/ unawareness of danger sign:

The decision to seek care in the current pregnancy is generally influenced by previous uncomplicated pregnancy. A relative of a deceased woman said that *“this was her 3*^*rd*^ *pregnancy, the other two were delivered at home, there was no problem. This pregnancy would also be happening without any hitch”.*

In another case a respondent narrated that *“She prepared morning food and was rolling bidi as usual. Suddenly she lied down on the bed and complained of abdomen pain. We thought pain would subside like last time; delivery would occur without any problem. When pain did not subside, we took her to the hospital”.*

##### Cultural beliefs and customs:

In rural areas elderly women who were in menopause are well acquainted with child bearing and pregnancy. *“For three days our daughter was unwell and then we called for Mahera Bibi (changed name - a village lady) from the neighbouring village. She was not at home. Waited for 3 hours and reached home. She observed that our daughter was suffering from internal bleeding and that’s why she was pale. Mahera Bibi suggested us to take her to hospital”* (Reported by a mother).

##### Previous unfavourable experience with the health system:

The unfavourable experience in the previous pregnancy with the government health care system has attributed to delay in seeking care. *“She developed pain on Monday night. We thought of taking her to the hospital in the next morning because in previous pregnancy she had to wait whole night in the ward of the hospital as there was no doctor” (*A relative of a deceased woman*).*

#### Delay in reaching appropriate medical facility

In 40 community-based verbal autopsies that we analysed 29 cases experienced delay in reaching an appropriate medical facility.

##### Transportation problem and financial constraints:

Narratives of the family members reveal that main transport has been auto-rickshaw, rickshaw, taxi and van- rickshaw. Most of the family members stated that they had to borrow money for transportation and medicine cost. Home to health facility centre, the transport cost was approximate 450 Rupees; transportation from first level to 2nd level costs was 1300 Rupees, and from level 2 to level 3 transportation costs was 2400 Rupees. The cost of transport from 3^rd^ level to study hospitals was 4500 Rupees.

##### Prolonged transportation:

Poor roads, visiting different health facilities and poor vehicle condition prolonged or aggravated the precarious health conditions.

**Fig. 2:**
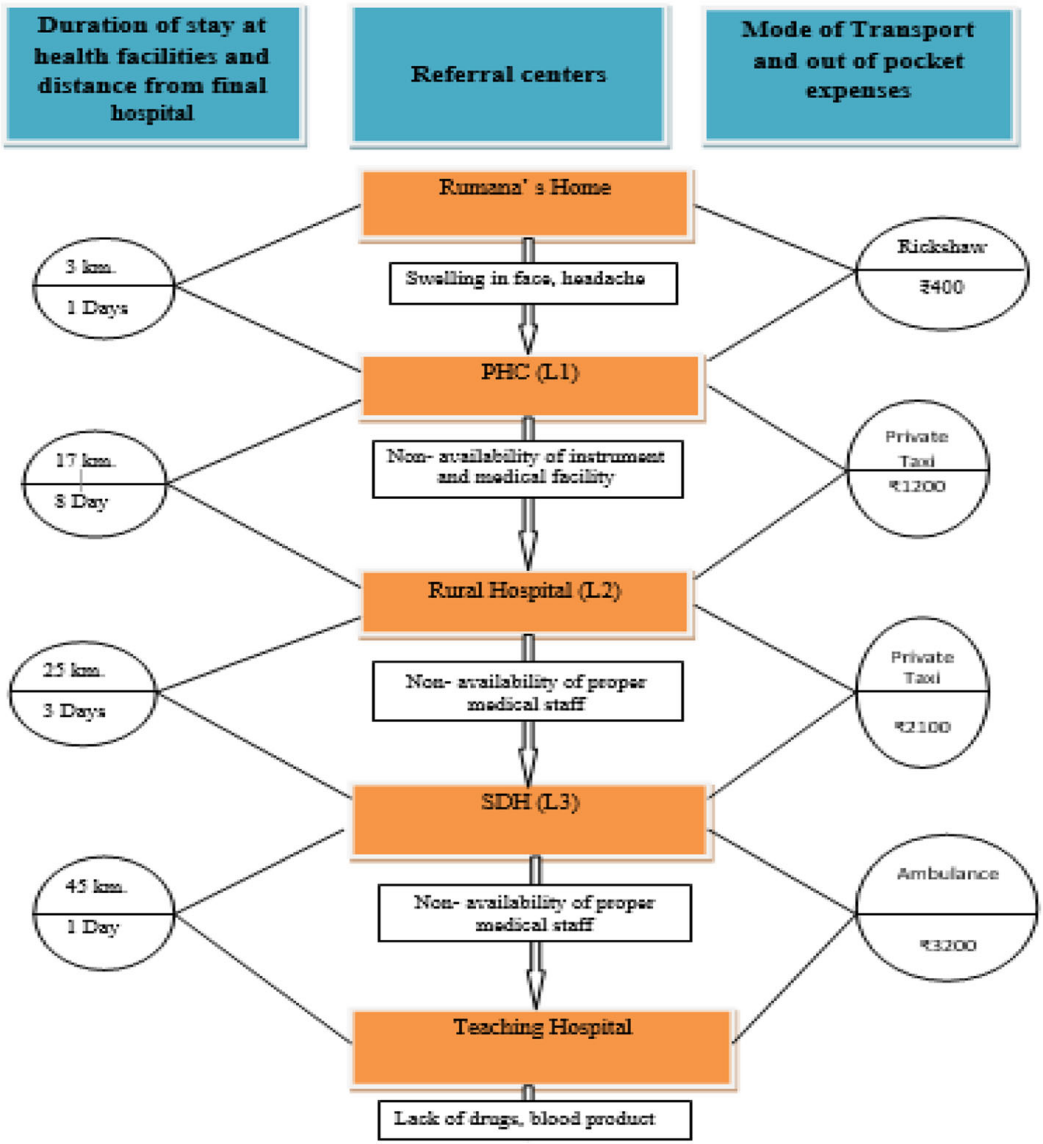
Story of Rumana (Changed Name)

*This can be highlighted by story of Rumana (changed name,* *Fig.* *2* *) and it can be illustrated in the following case*

Rumana, during her pregnancy complication, had to go through for 3 types of referral hospitals for receiving adequate comprehensive obstetric medical care (CMoC) at the study hospitals. This journey took 7 days and 8 hours to travel a distance of 90 Km, it took 9000 rupees out of pocket expenses to hire various modes of transport and buy medicines (Fig. 2).

#### Delay in receiving prompt and adequate treatment after reaching the hospital

Reasons for third delay are lack of blood transfusion, medical supplies and delay in start of treatment along with lack of medical staff.

A respondent explained *“when we reached hospital, they (doctors and nurses) said 2 units of blood is required. We went to the laboratory they said we have 1 bottle of blood we do not have two. You have to purchase one from outside. We paid 500 rupees to purchase 1 unit of blood.”*

In another case: a relative of a deceased woman narrated *“we reached at 8 PM. We had to wait for 2 hours. Doctors called the nurse over phone. Condition of the patient was serious and if we had to wait so long how would she survive?”.*

## Discussion

Thaddeus and Maine's "Three Delays" model is the recognition of the complex and interconnected nature of factors which are impediments to obtain high-quality maternal care for pregnant women and their families. This impediment plays an instrumental role in the road to maternal deaths [18, 19]. Considering the effectiveness of the "Three Delays" model, we have endeavoured to comprehend the barriers to seek obstetric care and contextualize them in the larger picture of maternal mortality in the major eclamptic maternal deaths region in the country.

The study revealed that the delay related to user factors (patients and/or their family) were the major contributors to maternal deaths as compared to the type 2 and type 3 delays. The verbal autopsies identified 35 women out of 40, who observed the delay in seeking care. The delay ranged between 2 hours to 3 days. Incorrect perception of the gravity of the circumstance resulted in delaying the decision to seek appropriate medical care until it was almost too late to deliver the instantly needed care has been highlighted in several studies [6, 7, 20]. Our findings on the contribution of type 1 delay to maternal deaths differ from those in the Malawi study by Mgawadere et al. [3], in which a high proportion of deceased women experienced type 3 delay. On the contrary, our results utterly analogous to the study done by Paul et al. [21] in the tertiary health care centre of Odisha where delay in deciding to seek care accounted for more than half of maternal deaths.

This study elucidated multiple reasons for the delay in seeking care which are generally barriers of the socio-cultural milieu. Barriers related to seeking medical care such as who will decide to seek care, inability to recognize the danger obstetric complications and symptoms of illness, birth preparedness, traditional beliefs, practices and customs continue to influence to delay in decision and departure to seek care [9, 22, 23]. Some previous studies also reported that the past poor experiences and expectations were the significant reasons for the type 1 delay [3, 22]. Proper and regular antenatal care during pregnancy is the determinant of health education to family to identify danger signs and ultimately helps in the decision-making process [7, 20]. Our results revealed a very low reporting of antenatal care which may answer the high contribution of type 1 delay to maternal deaths in the study. The influence of decision makers in determining to seek care have also reflected in several studies [20, 23]. It is important to recognize who is involved in the decision-making process regarding when and how to seek EmOc care; which is considered as a significant factor in the decision-making process. Studies from different parts of the world revealed male partners were the key decision maker for seeking care during gestation period [24, 25]. The delay in deciding to seek care is also influenced by the traditional “wait and see” approach. In “wait and see” approach pregnant women and their family members decide to take action only when conditions worsen or when a complication occurs.

The study explored that one-third of deceased women experienced a delay in reaching the first level health facility and resulted in second major contributor to maternal deaths [21, 26]. Along with other studies, our study also reported that availability of resources, financial consideration, transportation difficulties and long distance affected reaching the health facility [3, 7]. It is observed in many studies that public transportation may not be accessible at the time required or may take a considerable time to arrive at the health facility [27]. In our low socio-economic settings, earning daily wage may be prioritized over transportation expenses for reaching health facility at the time of complications [8, 28]. Pregnant women require health care services closer to them or promptly accessible affordable public transport as a solution for the type 2 delay.

In our study, delays related to supplier or provider side were less observed in 19 percent of maternal deaths. The third delay is relatively rarely reported as compared to first and second delays in the previous literature [28, 29]. Type 3 delay is directly indicative of suboptimal quality of care and it is also reflected in our study [6]. Shortages of essential drugs, lack of blood products or equipment or supplies were identified as significant barriers to receive adequate care in the study [3, 7]. Previously, research conducted in the study region reported that lack of anaesthetists at block level hospitals hindered in the process of emergency C-section [16].

The socio-demographic and obstetric indicators influenced on the delays occurred at different phases. The delay in seeking care as well as reaching first level health facilities were comparatively higher among older mothers, multiparas, multigravidas, rural women, referred cases, C-section and women who resided at distant areas from the studied hospitals. The odds ratio of first delay was significantly higher among women who belonged to older age group, multiparas and Muslim women as compared to their counterparts. This may be due to younger women, and multiparity mothers with earlier delivery experience made them unwilling to seek care from the facilities [3]. People have lack of awareness that the complications can happen in subsequent pregnancy. In addition to this, there was a lack of perception that even a normal pregnancy experience can end in complications in delivery and postpartum stages. The study conducted in India [30, 31], Bangladesh [20], Nepal [32] and Nigeria [3, 33] have shown an association of parity and age of death on delay in seeking care. Alongside with other studies, our study has also observed that the delay in deciding to seek care and reaching to first level health facility was significantly higher among Muslims mothers as compared to Hindus [34]. It is possibly due to the low socio-economic status and religious belief of Muslim community [35]. In many situations, Muslim family members prefer Muslim female doctors for regular medical check-ups during pregnancy and delivery because of their religious faith [10, 35]. The distance from final hospital also contributed in delay in reaching health facility in the study. Due to transportation logistics, the women and their family members staying far generally believe in the “wait and see” approach to decide when to reach the health facility [3, 20].

The limitations of this study can be illustrated on two grounds: data-based and theoretical model based. We have collected data based on what medical professionals have documented in hospital records. Many occasions, the investigators or nodal officers are unaware of what happened before the patient arrived at that health setup. So, the documentation of the first two types of delays may not be accurate. On the other hand, the “Three Delays” model developed by Thaddeus and Maine has not discussed the concealment and refusal of treatment by patients and may warrant further exploration. This model is mainly utilized to describe why delays in obtaining emergency obstetric care occurred; it does not address issues related to access to antenatal or postnatal care. The framework needs rethink to account the aforementioned issues.

In spite of these limitations, this study provides a unique dimension in the field of maternal and child health research by employing facility and community based maternal death review approaches in one platform. This endeavour of applying a mixed approach to examine the demand-side and supplier-side barriers on maternal mortality is rarely being observed in previous studies. The present study uses both facility and community-based death review approaches which not only provide the information on service provider’s side barriers but also provides an insight into what kind of delays happened from the family side. The study suggests that pregnant women demand the establishment of separate eclampsia units at lower levels health facilities to reduce the maternal deaths as we have seen eclampsia-related maternal deaths mostly occurred among type-1 and type-2 of delays experienced women. Public-private partnership model or multi-sectoral approach can be further recommended to combat the adverse maternal health outcomes in West Bengal.

## Conclusions

The study found that the delays related to household and transport were the major contributors of maternal deaths in the study region. Recognition of danger signs, illiteracy and ignorance, knowledge and attitude about seeking medical care, arranging transport and financial constraints were the main barriers of delay in seeking care and reaching facility. The first type of delay was significantly higher among older aged, multiparous and Muslim women. Therefore, to prevent the maternal deaths effectively will demand action in three areas: first, expanding the coverage of functioning referral networks, both community and service provider based, for obstetric emergencies; secondly, raising awareness regarding maternal complications, danger sign and finally, improving the ability of existing medical institutions to deliver effective and high-quality health care.

## Data Availability

The datasets analyzed for this study is available with corresponding author which can be accessed on reasonable request.

## Abbreviations

ANC: Antenatal care
ANM: Auxiliary nurse midwife
BEMoC: Basic emergency obstetric care
BHTs: Bed heat tickets
CBMDR: Community based maternal death review
CEmOC: Comprehensive emergency obstetric care
FBMDR: Facility based maternal death review
IECHR: Institutional Ethics Committee for Human Research
KMC&H: Medical college & hospital, kolkata
MMC&H: Malda medical college & hospital
MMR: Maternal mortality ratio
MoHFW: Ministry of health and family welfare
PET: Pre eclamptic toxaemia
SC: Sub centre
WHO: World health organization
